# Subgroup Analysis in Burnout: Relations Between Fatigue, Anxiety, and Depression

**DOI:** 10.3389/fpsyg.2016.00090

**Published:** 2016-02-04

**Authors:** Arno van Dam

**Affiliations:** ^1^GGZ WNB Mental Health Institute, Research and InnovationHalsteren, Netherlands; ^2^Tranzo Scientific Center for Care and Welfare, Tilburg UniversityTilburg, Netherlands

**Keywords:** burnout, depression, chronic fatigue syndrome, anxiety, fatigue, nosological classification, diagnosis, cluster analysis

## Abstract

Several authors have suggested that burned out patients do not form a homogeneous group and that subgroups should be considered. The identification of these subgroups may contribute to a better understanding of the burnout construct and lead to more specific therapeutic interventions. Subgroup analysis may also help clarify whether burnout is a distinct entity and whether subgroups of burnout overlap with other disorders such as depression and chronic fatigue syndrome. In a group of 113 clinically diagnosed burned out patients, levels of fatigue, depression, and anxiety were assessed. In order to identify possible subgroups, we performed a two-step cluster analysis. The analysis revealed two clusters that differed from one another in terms of symptom severity on the three aforementioned measures. Depression appeared to be the strongest predictor of group membership. These results are considered in the light of the scientific debate on whether burnout can be distinguished from depression and whether burnout subtyping is useful. Finally, implications for clinical practice and future research are discussed.

## Introduction

Burnout is a stress-related syndrome characterized by exhaustion, cynicism, and reduced perceived job competence (PJC; Schaufeli and Enzmann, 1998; Maslach et al., 2001). Burnout patients report many symptoms such as reduced job satisfaction, physical complaints, fatigue, sleep disturbances and impaired cognitive performance (Schaufeli and Enzmann, 1998; Maslach et al., 2001; Taris, 2006; Schmidt et al., 2007). Exhaustion, the feeling of depletion due to effort spent at work (Maslach et al., 1996; Schaufeli and Enzmann, 1998), is generally regarded as the most distinctive feature of the burnout syndrome (Brenninkmeijer and Van Yperen, 2003; Shirom, 2003; Bekker et al., 2005; Kristensen et al., 2005) and appears to overlap considerably with the concept of fatigue (Huibers et al., 2003; Schaufeli and Taris, 2005; Leone et al., 2007). In 1970s of the 20th century, the term burnout was introduced in the scientific literature to describe the emotional exhaustion of workers in education, health care, and service occupations. Today the term is used to indicate exhaustion and loss of motivation as a result of work in general. Both paid work and unpaid work like the work of informal carers, students, housewives and volunteers fall within the definition (Schaufeli, 2007). There is discussion in the literature on burnout whether the attribution of exhaustion to work, or stress in general should be part of the definition (Kristensen et al., 2005; Öhman et al., 2007; Bianchi et al., 2014b). The definition of burnout is for example very similar to that of chronic fatigue syndrome (CFS) with regard to fatigue and restraints on daily functioning. Burnout, however, can be distinguished from CFS by the attribution of fatigue to work instead of somatic factors (Hoogduin et al., 2001; Huibers et al., 2003). The scientific debate on whether it is useful to make such distinctions between disorders based on attribution of symptoms has not reached consensus among researchers and clinicians. This discussion is complicated by the fact that burnout is not recognized in the main international disease classification systems. Therefore, there is no international consensus on how to diagnose burnout, resulting in national differences in the conceptualisation and classification of the syndrome (Schaufeli, 2007). Burnout symptoms and depressive symptoms also seem to be interrelated to a certain degree (Schaufeli and Enzmann, 1998). Several studies found that the phenomenological overlap between burnout and major depressive disorder appeared to be rather small (Leiter and Durup, 1994; Glass and McKnight, 1996; Schaufeli et al., 2001; Toker et al., 2005), but the results of other recent studies suggest that there may be considerable overlap between burnout and depression (Bianchi et al., 2014a, 2015b). A reason for these contradicting findings may be that there are subgroups of burnout patients and subgroups of patients with major depression. In the DSM there are different categories of major depression, for example melancholic depression, catatonic depression and atypical depression. It is possible that burnout overlaps with some of these recognized subgroups or that intermediate levels of burnout cluster with intermediate levels of depressive symptoms. These intermediate levels of burnout may for example cluster with subclinical levels of depression. Martin et al. (1996) found that subclinical depression was related to reduced performance at work. Bianchi et al. (2014a) suggest that the subgroup atypical depression may account for a substantial part of the burnout depression overlap. The recognized subgroups of depression, however, do also appear not to be homogeneous (Jang et al., 2004; Lux and Kendler, 2010; Goldberg, 2011).

This criticism on the classification of major depression among other disorders is part of a larger discussion in psychiatry. An increasing number of authors argue that our current way of classification of psychiatric diseases is not sufficient (Harvey et al., 2004; Bentall et al., 2009; McLaughlin and Nolen-Hoeksema, 2011). Although the DSM approach of classification has undeniably increased our knowledge and expertise regarding psychopathology, it also suffers from serious limitations. First, there is the problem of comorbidity. About half of all individuals with a particular mental disorder, also have another mental disorder in addition (Brown and Barlow, 1992). Second, common risk and vulnerability factors underlie different psychological disorders (Harvey et al., 2004). Third, the same psychological and pharmacological interventions are effective in several psychological disorders (Tsao et al., 2002).

These phenomena may also apply for syndromes like depression, CFS, and burnout. Several studies showed high comorbidity rates for depression, CFS, and burnout (Afari and Buchwald, 2003; Bianchi et al., 2015a,b). Stress is recognized as a preceding factor for depression, CFS as well as burnout (Pall, 2001; Plieger et al., 2015) and reduced self-efficacy as maintaining factor for all three syndromes (van Dam et al., 2013; Vollmayr and Gass, 2013; Knoop and Wiborg, 2015). Cognitive behaviour therapy (CBT) appears to be an effective treatment for depression, CFS, and burnout (Stenlund et al., 2009; Tummers et al., 2012; Cuijpers et al., 2013). Many researchers and clinicians believe that a transdiagnostic approach of psychiatric disorders is more promising in order to understand and treat these disorders (Harvey et al., 2004; Mansell et al., 2009). The focus of study and treatment should be more directed to specific symptoms and their function than on the classification of syndromes. For example, fatigue is a symptom accompanying a variety of disorders like depression, burnout, generalized anxiety disorder, and CFS. Theoretical models on the function of fatigue (Boksem and Tops, 2008) and specific treatments aimed at reducing fatigue (Tummers et al., 2012) may be applicable in a variety of disorders.

Several authors have suggested that burnout patients also do not form a homogeneous group and that there are subtypes (Demerouti et al., 2005; Tops et al., 2007; Oosterholt et al., 2014), or that the symptomatology of burnout may be different for different stages of the burnout syndrome (Edelwich and Brodsky, 1980; Golembiewski and Munzenrider, 1988; Golembiewski and Boss, 1991). Tops et al. (2007) distinguish two subtypes of burnout patients based on psychophysiological differences. A group with increased prolactine levels showed high levels of task engagement as opposed to a group with low prolactine levels who showed low task engagement. Both groups had elevated levels of fatigue, depression and anxiety but differed from each other on the tendency to express and share emotions and feelings. The high prolactin burnout subjects were responsive to cortisol supplements, resulting in an increase in vigor and decrease of fatigue whereas the low-level prolactine group was not responsive to cortisol supplements. Van Dam et al. (2015a) described similar subgroups of burnout patients. In a study on task performance strategies in burnout patients, they found, in the same line as Tops et al. (2007), that there was a group showing high task engagement and another group with poor task performance (van Dam et al., 2013). The existence of an active subgroup with high levels of task engagement and a passive subgroup with low task engagement is in line with phase models of burnout (Edelwich and Brodsky, 1980) in which an early phase of burnout is characterized by trying to maintain high levels of task-performance despite fatigue. In this phase individuals experience stress and anxiety due to failing coping strategies. High levels of task engagement do not seem to be rewarding. In the final phase of burnout, which is characterized by passivity and fatigue, individuals gave up and did not try to cope with (working) demands anymore. Van Dam et al. (2015a) compared this stage with the learned helplessness syndrome as proposed by Seligman (1975) as an underlying process for depression. Learned helplessness theory is the view that clinical depression and related mental illnesses may result from real or perceived absence of control over the outcome of a situation. This may be an explanation for the considerable overlap between burnout and depression that is found in some studies. The findings in these studies suggests; however, that there may also be a subgroup of burnout patients who are in a relative early stage of burnout characterized by high levels of stress and anxiety. They will probably show less overlap with depression. In order to test this hypothesis, level of fatigue, level of depression and level of anxiety were measured in a group of clinically diagnosed burnout patients. Subsequently a two-step cluster analyses was performed. It was hypothesized that the analyses would reveal two subgroups, both subgroups showing equally high levels of fatigue because fatigue is the central feature of burnout but differing from each other in levels of anxiety and depression, resulting in a mainly anxious burnout group and a mainly depressed burnout group.

## Materials and Methods

For this study, data of burnout patients who participated in studies on cognitive performance and appraisal of fatigue in burnout were used (van Dam et al., 2011; Van Dam et al., 2015b). Approval for the studies was obtained from the Ethical Committee (ECG) of the Faculty of Social Sciences of Radboud University Nijmegen in the Netherlands.

### Participants

Burnout patients (*N* = 113) were recruited from mental health centers in the Netherlands, where they asked for treatment for their symptoms. The mean age was 44.4 (*SD* = 9.0) years old, 60 (53.1 %) were men, 10 (9%) had a low level of education, 51 (45%) a middle level and 52 (46%) had a high level of education. We provided therapists of various mental health organizations with brochures about the research projects and asked them to give these to their patients. When patients wanted to participate, they signed an informed consent form and sent it to the researcher. Subsequently, the patient was invited for a semi-structured interview. For inclusion, patients had to meet: (1) the validated cut-off points (Brenninkmeijer and Van Yperen, 2003) for severe burnout of the Dutch version (Utrecht BurnOut Scale-A, UBOS-A; Schaufeli and van Dierendonck, 2000) of the Maslach Burnout Inventory (MBI) General Survey (Maslach et al., 1996): exhaustion ≥2.20 and either cynicism ≥2.00 or PJC ≤3.67; (2) the cut-off point for prolonged fatigue of the Checklist Individual Strength (CIS; 76) (Bültmann et al., 2000); (3) the criteria for the proposed psychiatric equivalents of clinical burnout, namely the ICD-10 (World Health Organisation [WHO], 1994) criteria for work-related neurasthenia (Schaufeli and Enzmann, 1998; Schaufeli et al., 2001); and (4) the DSM-IV (American Psychiatric Association [APA], 2000) criteria for unspecified somatoform disorder with prolonged fatigue as the main symptom (Hoogduin et al., 2001). In DSM-5 burnout can be diagnosed with somatic symptom disorder (van Dam et al., in press).

Unspecified somatoform disorder was established with the Dutch translation (Overbeek et al., 1999) of the Mini International Neuropsychiatric Interview (Sheehan et al., 1998), and work-related neurasthenia with a semi-structured interview checking ICD-10 criteria for work-related neurasthenia (World Health Organisation [WHO], 1994). Three patients also met the criteria of simple phobia as a secondary diagnosis and 1 patient met the criteria of a panic disorder as secondary diagnosis.

### Measures

Severity of burnout symptoms was assessed with the Dutch version of the MBI General Survey (Maslach et al., 1996), referred to as the Utrecht BurnOut Scale-A (UBOS-A; Schaufeli and van Dierendonck, 2000), which is composed of 15 questions answered on a 7-point Likert scale (0: never, 6: every day) measuring three dimensions: (1) exhaustion (E), (2) cynicism (C), and (3) PJC. High scores on exhaustion and cynicism and low scores on PJC is an indication of burnout. Reliability and validity of the UBOS are good. Cronbach’s alpha’s are: E = 0.84, C = 0.71, PJC = 0.72 (Schaufeli and van Dierendonck, 2000).

The level of fatigue was assessed with the Dutch version (Vercoulen et al., 1999) of the Checklist Individual Strength (CIS; Vercoulen et al., 1994). The 20 checklist items measures four subscales: subjective feelings of fatigue and physical fitness (SF), activity level (A), motivation (M), and concentration (C) during the previous 14 days. The total score is an indication for general fatigue. Reliability and validity of the CIS are high, Cronbach’s alpha for the CIS is 0.90 (Vercoulen et al., 1994). Reliability of the subscales is also good: Cronbach’s alpha’s are: SF = 0.99, A = 0.87, M = 0.83, C = 0.92 (Vercoulen et al., 1994).

Level of depressive symptoms was measured with the 16-item depression subscale of the Dutch adaptation (Arrindell and Ettema, 2005) of the Symptom Checklist (SCL-90; Derogatis, 1977). The reliability and validity of the SCL-90 are high, Cronbach’s alpha of Depression subscale ranges from 0.88 to 0.94 (Arrindell and Ettema, 2005).

The level of anxiety was measured with the anxiety subscale of the Dutch adaptation (Arrindell and Ettema, 2005) of the Symptom Checklist (SCL-90; Derogatis, 1977). The 10 items measure general anxiety. The reliability and validity of the SCL-90 are high, Cronbach’s alpha’s for the Anxiety subscale of the SCL-90 range from.87 to.92 (Arrindell and Ettema, 2005).

## Results

In order to explore relations between burnout, fatigue, depression, and anxiety, basic correlations and correlations corrected for attenuation (Cohen et al., 2003), between the subscales of the MBI, the subscales of the CIS and the depression and anxiety subscales of the SCL-90 were calculated. See **Table 1.**

**Table 1:** Means, SDs, Cronbach’s alpha (α) in this study, and correlations (correlations corrected for attenuation are in *italic*) between subscales of the Maslach Burnout Inventory (MBI); E: exhaustion; C: cynicism; PJC: perceived job competence; the anxiety (A) and depression (D) subscales of the symptom checklist-90, the subscales of the checklist individual strength (CIS); SF, subjective fatigue; C, concentration; M, Motivation; A, Activity and the total score GF, general fatigue.

	*M (SD)*	α	1	2	3	4	5	6	7	8	9	10
1 MBI- E	4.95 (0.99)	0.87		0.48^∗∗^	-0.36^∗^	0.21	0.38^∗^	0.42^∗∗^	0.18	0.33^∗^	0.56^∗∗∗^	0.60^∗∗∗^
2 MBI- C	3.66 (1.29)	0.76	*0.59^∗∗^*		-0.41^∗∗^	0.10	0.16	0.17	0.35^∗^	0.15	0.07	0.29
3 MBI- PJC	3.23 (1.00)	0.63	-*0.49^∗^*	-*0.59^∗∗^*		-0.29	-0.41^∗∗^	-0.16	-0.30	-0.40^∗∗^	-0.08	-0.37^∗^
4 SCL-90- A	21.11 (7.74)	0.79					0.66^∗∗∗^	0.24^∗∗^	0.16	0.25^∗∗^	0.03	0.25^∗∗^
5 SCL-90- D	38.81 (11.29)	0.90	*0.43^∗^*		-*0.54^∗∗^*	*0.78^∗∗∗^*		0.37^∗∗∗^	0.38^∗∗∗^	0.39^∗∗∗^	0.26^∗∗^	0.49^∗∗∗^
6 CIS- SF	44.72 (9.49)	0.84	*0.49^∗∗^*			*0.29^∗∗^*	*0.43^∗∗∗^*		0.45^∗∗∗^	0.44^∗∗∗^	0.24^∗^	0.81^∗∗∗^
7 CIS- C	26.87 (6.30)	0.72		*0.47^∗^*			*0.47^∗∗∗^*	*0.58^∗∗∗^*		0.45^∗∗∗^	0.23^∗^	0.72^∗∗∗^
8 CIS- M	18.59 (6.08)	0.82	*0.39^∗^*		-*0.56^∗∗^*	*0.31^∗∗^*	*0.45^∗∗∗^*	*0.53^∗∗∗^*	*0.59^∗∗∗^*		0.27^∗∗^	0.73^∗∗∗^
9 CIS- A	13.86 (6.48)	0.64	*0.78^∗∗∗^*				*0.34^∗∗^*	*0.33^∗^*	*0.34^∗^*	*0.37^∗∗^*		0.60^∗∗∗^
10 CIS- GF	104.04 (20.42)	0.80	*0.72^∗∗∗^*		-*0.52^∗^*	*0.31^∗∗^*	*0.71^∗∗∗^*	*0.99^∗∗∗^*	*0.95^∗∗∗^*	*0.90^∗∗∗^*	*0.83^∗∗∗^*	


*^∗^*p* < 0.05; ^∗∗^*p* < 0.01; ^∗∗∗^*p* < 0.001.*

The correlations revealed many signific ant relations between the study variables. Especially depression and anxiety appeared to be strongly related, exhaustion appeared to be related to general fatigue, cynicism, and depression. General level of fatigue was related to depression and anxiety, the CIS subscales were closely related to each other. PJC was negatively related to exhaustion, cynicism, depression, and general level of fatigue.

A two-step cluster analysis was conducted with fatigue (CIS), depression (Scl-90), and anxiety (Scl-90) as variables. In the two-step cluster analysis, the number of clusters were not determined in advance, but determined automatically by SPSS on basis of the best fit. Model fit indicated by the average silhouette of cohesion and separation was 0.5, which is considered as good (Kaufman and Rousseeuw, 1990). The analysis revealed a model with two clusters. The clusters differed from each other on all three measures in the same way. The scores on the measures in cluster one suggest mild psychopathology and the scores on the measures in cluster two suggest severe psychopathology.

Comparisons of means revealed that the burnout patients in the two clusters differed from each other on general level of fatigue *F*(1,112) = 41.7, *p* < 0.001, level of depression *F*(1,112) = 140.9, *p* < 0.001, and level of anxiety *F*(1,112) = 92.1, *p* < 0.001, but did not differ from each other regarding, gender, age, and level of education. The most important predictor for group membership appeared to be depression (1.0), followed by anxiety (0.75) and fatigue as least important (0.41). Compared to healthy norm groups, level of fatigue was high in both groups (Vercoulen et al., 1999). The group with severe symptoms had similar levels of fatigue as patients with CFS (Vercoulen et al., 1999). With regard to anxiety and depression, the group with mild symptoms scored in the range between the healthy and the psychiatric population, the group with severe symptoms scored in the same range on depression and anxiety levels as the psychiatric population (Arrindell and Ettema, 2005). An additional analysis, which also included the MBI, subscales in the analysis revealed similar results. Model fit indicated by the average silhouette of cohesion and separation was.4, which is considered as fair (Kaufman and Rousseeuw, 1990). The analysis also revealed a model with two clusters. The clusters differed from each other on all measures in the same way. The scores on the measures in cluster one suggest mild psychopathology and the scores on the measures in cluster two suggest severe psychopathology. Also in this analysis depression appeared to be the most important predictor for group membership (1.0), followed by anxiety (0.60), PJC (0.60), exhaustion (0.57), general fatigue (0.37) and cynicism as least important (0.35). The means and SDs on the measures for the two clusters are presented in **Table 2.**

**Table 2:** Demographic variables, Means, SDs, effect size of group difference, and predictor importance for cluster membership of the measures for the two clusters.

	Cluster 1	Cluster 2	Effect-size	Predictor
	Mild Burnout	Severe Burnout	Cohen’s *d*	Importance for cluster membership
	(*N* = 58)/51%	(*N* = 55)/49%		
Gender: men (%)	34 (58.6%)	26 (47.3%)		
Age (SD)	44.2 (8.5)	44.5 (9.7)		
Educational level (%)				
Low	4 (7%)	6 (11%)		
Middle	27 (47%)	24 (44%)		
High	27 (47%)	25 (45%)		
1 SCL-90- D^∗∗∗^	30.6 (5.8)	47.4 (9.0)	2.25	1.0
4 SCL-90- A^∗∗∗^	16.1 (4.4)	26.4 (6.9)	1.81	0.60
3 MBI – PJC^∗^	3.6 (.8)	2.9 (1.0)	0.78	0.60
1 MBI- E^∗^	4.6 (1.1)	5.2 (0.8)	0.63	0.57
10 CIS- GF^∗∗∗^	93.7 (20.3)	114.9 (13.9)	1.22	0.37
2 MBI- C	3.4 (1.5)	3.8 (1.1)	0.31	0.35
6 CIS- SF^∗∗∗^	40.7 (10.1)	49.0 (6.6)	0.98	
7 CIS- C^∗∗∗^	24.3 (6.7)	29.6 (4.5)	0.76	
8 CIS- M^∗∗∗^	16.1 (5.8)	21.2 (5.2)	0.93	
9 CIS- A^∗^	12.6 (6.8)	15.2 (6.0)	0.41	


*^∗^*p* < 0.05; ^∗∗^*p* < 0.01; ^∗∗∗^*p* < 0.001.*

*Maslach Burnout Inventory (MBI); E: exhaustion; C: cynicism; PJC: perceived job competence; the anxiety (A) and depression (D) subscales of the symptom checklist-90, the subscales of the checklist individual strength (CIS); SF: subjective fatigue; C: concentration; M: Motivation; A: Activity and the total score GF: general fatigue. Cohen (1988) suggested that *d* = 0.2 can be considered a ‘small’ effect size, 0.5 represents a ‘medium’ effect size and 0.8 a ‘large’ effect size.*

In order to investigate whether the differences between the subgroups are clinically relevant we calculated effect sizes (Cohen’s *d*) for the main variables (see **Table 2**). The effect sizes for all variables were moderate to large (Cohen, 1988). The effect size for depression was the largest, followed by anxiety and fatigue.

In order to analyze whether the split in the data is done in terms of the distributional properties of the data we performed Shapiro–Wilk’s statistics for the main variables in both the original dataset and in the subgroups. We expected that the main variables would be distributed normally in the subgroups but not in the initial dataset. The analysis revealed that in the initial dataset depression and fatigue were not distributed normally (depression: W = 0.96, *p* < 0.01; fatigue: W = 0.97, *p* < 0.01) and were distributed normally in the two subgroups (subgroup 1: depression: W = 0.98, *p* = 0.40; fatigue: W = 0.98, *p* = 0.29; subgroup 2: depression: W = 0.97, *p* = 0.20; fatigue: W = 0.98, *p* = 0.33). Anxiety and exhaustion are not distributed normally in the initial dataset (anxiety: W = 0.93, *p* < 0.001; exhaustion: W = 0.88, *p* < 0.001) as well as in the subgroups (subgroup 1: anxiety: W = 0.87, *p* < 0.01; exhaustion: W = 0.89, *p* < 0.05; subgroup 2: anxiety; W = 0.93, *p* < 0.001; exhaustion; W = 0.89, *p* < 0.01). Cynicism and PJC are distributed normally in the initial dataset (cynicism: W = 0.97, *p* = 0.39; PJC: W = 0.96, *p* = 0.11) as well as in the subgroups (subgroup 1: cynicism: W = 0.96, *p* = 0.52; PJC: W = 0.25, *p* = 0.29; subgroup 2: cynicism: W = 0.97, *p* = 0.75; PJC: W = 0.96, *p* = 0.52).

In order to test whether the structure of the subgroups differed from each other and from the initial dataset we calculated correlations between the main variables in the subgroups and compared them with each other and the correlations in the initial dataset (**Table 1**). Depression and anxiety are correlated in both the subgroups (subgroup 1, *r* = 0.26 *p* < 0.05; subgroup 2, *r* = 0.34, *p* < 0.05) and in the initial sample (*r* = 0.66, *p* < 0.001). Exhaustion and fatigue are correlated in both the subgroups (subgroup 1, *r* = 0.50, *p* < 0.05; subgroup 2, *r* = 0.60, *p* < 0.01) and in the initial sample (*r* = 0.60, *p* < 0.001). In the initial sample but not in both subgroups a correlation between depression and fatigue (*r* = 0.49, *p* < 0.001) and a correlation between depression and exhaustion (*r* = 0.38, *p* < 0.05) was found.

## Discussion

Based on previous studies and the literature on burnout it was expected that in a population of burnout patients, two subgroups could be distinguished: a mainly anxious burnout group and a mainly depressed burnout group with equal levels of fatigue. The results confirmed that two subgroups of burnout patients could be distinguished. The difference between the subgroups; however, was not as expected. The groups differed with regard to symptom severity, resulting in a group with mild symptoms and a group with severe symptoms on all measures (burnout, fatigue, depression, and anxiety). The finding that depression and fatigue were not normally distributed over the initial dataset and were distributed normally in the two subgroups supports the hypothesis that burnout patients do not form a homogeneous group and comprises two subgroups. The large effect sizes for subgroup differences between depression and anxiety suggest that these differences are clinically relevant. The relations between the study-variables were comparable in both subgroups which indicate that the subgroups only differed from each other regarding symptom severity and not regarding structure. Correlations between depression and exhaustion and depression and fatigue were present in the initial sample but not in the subgroups. This finding suggests that in the subgroups, fatigue and depression are not just two related aspects of the burnout syndrome. In the subgroups, level of fatigue is not dependent of level of depression and vice versa. A high level of depression seems to be an additional condition in the severe burnout group.

The mild symptom group seems to be a subclinical group (Arrindell and Ettema, 2005) with fatigue as most prominent symptom, although level of fatigue is below the level of fatigue compared to level of fatigue in patients suffering from CFS (Vercoulen et al., 1999) and from patients suffering from depression (Van Dam et al., 2015b). The group with severe symptoms is comparable with the average psychiatric population (Arrindell and Ettema, 2005) and with regard to fatigue with patients suffering from CFS (Vercoulen et al., 1999) and patients with depression (Van Dam et al., 2015b). The finding that the levels of anxiety and depression are psychiatrically relevant in the group with severe burnout suggests that burnout can be a serious burden for patients and that there may be overlap with anxiety and depressive disorders. The severe burnout group will probably overlap to some degree with the DSM-5 category of major depression with anxious distress but probably not coincide completely because a diagnosis of major depression according to DSM-IV-TR was excluded for all participants in the sample.

The differentiation between mild and severe burnout is not new in the literature on burnout. For more than three decades authors have argued that a distinction should be made between a mild form of work stress related psychological h that does not prevent an employee from working and a serious clinically relevant pattern of personal distress and diminished performance which is the end stage of the burnout process. These two types of burnout are mostly referred to as clinical and non-clinical burnout (Paine, 1982; Schaufeli et al., 2001; Brenninkmeijer and Van Yperen, 2003; Oosterholt et al., 2014). The clinical group being referred to psychological treatment due to symptom severity and inability to work and diagnosed by a clinician with burnout. The non-clinical burnout group comprises relative healthy employees, who report burnout symptoms but are not on sick leave and don’t seek treatment for their symptoms.

In the current study, however, all patients were diagnosed with burnout by a clinician and all patients fulfilled the criteria of clinical burnout as proposed in literature (Schaufeli et al., 2001; Brenninkmeijer and Van Yperen, 2003). However, also in this group of clinically diagnosed burnout patients, still a group with mild and a group with severe symptoms could be distinguished. This finding suggests that the current methods of diagnosing burnout are not sufficient to distinguish clinical from non-clinical burnout. Although the existence of a subgroup of burnout patients with mild symptoms and a group of burnout patients with severe symptoms may be in line with phase models of burnout (Edelwich and Brodsky, 1980), it is rather problematic that the current definitions of burnout comprise different groups. Relations between diagnosis, treatment and prognosis of treatment may become unclear and even contradictory.

In order to reduce these problems, attempts to improve definitions and categorisations of burnout (Kleijweg et al., 2013; Bianchi et al., 2014a) should be encouraged. Although the classification of disorders can increase our knowledge and expertise regarding psychopathology, it also has limitations. The existence of subgroups in diagnostic categories is regarded as a major shortcoming of current classification systems of psychiatric disorders (Radden, 1994; Follette and Houts, 1996; Goldberg, 2011). There is considerable heterogeneity in the presentations of symptoms within disorders. This criticism does not only apply to syndromes such as burnout that are not recognized in the main classification systems, but also to DSM-categorized syndromes such as major depression (Jang et al., 2004; Lux and Kendler, 2010; Goldberg, 2011). Many researchers and clinicians believe that a transdiagnostic approach to psychiatric disorders is promising in order to understand and treat these disorders (Harvey et al., 2004; Mansell et al., 2009). Research on predisposing, precipitating and maintaining factors like stress, sleep disturbance, rumination and learned helplessness and the function of symptoms like fatigue and anxiety may lead to a better understanding of psychopathology and the application of tailor-made interventions within diagnostic groups.

The finding that depression is the most important predictor for group membership suggests that the onset of depressive symptoms may be an important factor in exacerbation of symptoms in burnout. This is in line with studies that show that burnout may evolve to depression (Hakanen et al., 2008; Toker and Biron, 2012; van Dam et al., 2013). However, other studies have shown that burnout does not necessarily lead to depression, because burnout and depression overlap to a large extent from the start (Bianchi et al., 2015c) or that burnout and depression develop in tandem (Ahola et al., 2014). In the present study we also found burnout and depression to be related, although to a lesser extent than in Bianchi et al.’s (2015c) study.

The very large effect size of the difference between the subgroups on depression (the largest effect size compared to the other measures), underlines the importance of depression for subgroup membership. The differences between the subgroups on the burnout measures were moderate to large, which is much smaller than the differences found in depression, anxiety and fatigue between the two subgroups.

As suggested by Bianchi et al. (2014a) burnout patients may benefit from treatments designed for patients with major depression. This may especially the case for burnout patients whose symptoms appear to become chronic despite CBT (van Dam et al., 2012). Although several studies found a minor overlap between depression and burnout, the effectiveness of this type of treatment might still be efficacious in burnout patients, regardless of overlap between depression and burnout.

Besides the relation between burnout and depression, this study also showed a strong relation between depression and anxiety. This finding is in line with many studies (Löwe et al., 2008; Lamers et al., 2011) that show high levels of comorbidity between depression and anxiety.

Several limitations of the present study need to be addressed. First, although the criteria used in the current study to diagnose burnout have been broadly used in Dutch mental healthcare, they are not recognized in the main international disease classifications (DSM-5 and ICD-10) and therefore remain non-consensual. The generalizability and comparability of our results may therefore be limited to the+ Dutch context. Second, because there are no control groups in this study we do not know whether the subgroups we found are burnout-specific. The absence of well controls has probably led to smaller correlation coefficients between the study-variables. Third, the operationalization of anxiety and depression with the SCL-90 subscales may have affected the comparability of this study with other correlational studies in which specific depression inventories like the BDI-II are used. Although the SCL-90 depression subscale is highly correlated with the BDI-II (Steer et al., 1997) it cannot be ruled out that the use of the SCL-90 subscale resulted in lower correlations between burnout and depression compared to other studies (Bianchi et al., 2015c). A fourth limitation is the high correlations between some measures in this study, especially depression and anxiety. The high correlations indicate that the measures we used may not have been specific enough to distinguish subgroups. Questionnaires that measure more specific mechanisms like learned helplessness or self-efficacy may be more suitable for subgroup analysis in burnout. A fifth limitation is the cross-sectional design of the study. This design makes it impossible to conclude whether the subgroups we found are categorically different diagnoses of burnout, a milder form where individuals show engagement with work and a more severe form in which work engagement is limited or that the milder form develops into the more severe form.

## Conclusion

The results of this study showed that even in a group of burnout patients who are carefully diagnosed by clinicians, subgroups could be distinguished. This suggests that the diagnostic criteria for clinical burnout are problematic. The current way of diagnosing burnout includes patients with mild symptoms and a group with severe symptoms. The mild symptom group seems to be a subclinical group with fatigue as most prominent symptom. The group with severe symptoms is comparable with the psychiatric population with regard to fatigue, depression, and anxiety.

## Author Contributions

AvD was responsible for the data collection, the studydesign, analysis and interpretation of the results.

## Conflict of Interest Statement

The author declares that the research was conducted in the absence of any commercial or financial relationships that could be construed as a potential conflict of interest.
